# In-depth study of road accidents in Florence: understanding the biomechanical effects in major trauma involving vulnerable road users

**DOI:** 10.1186/cc14391

**Published:** 2015-03-16

**Authors:** A Franci, S Piantini, M Pierini, A Peris, M Mangini

**Affiliations:** 1 A.O.U. Careggi, Firenze, Italy; 2 University of Florence, Italy

## Introduction

Road accidents are the leading cause of death for young people, 50% being represented by vulnerable road users (VRU) (pedestrians, cyclists). In-depth accident studies assess the consequences of lack of use of safety devices and the need to develop new ones. Since 2009 a permanent team (physicians and engineers) has performed in-depth studies on road trauma admitted to our ICU [1].

## Methods

The team studied 52 VRU crashes that occurred in an urban area. The clinical data included an injury assessment using total body CT scan, Injury Severity Score (ISS), Abbreviated Injury Score (AIS), ICU and hospital length of stay and outcome score. Engineers collect data onsite with the partnership of the police, and assess the dynamics of the vehicles with the most advanced reconstruction techniques. Medical and engineering data were cross-matched during the correlation process. Injuries suffered by each person were related to specific impact objects.

## Results

**Figure 1:**
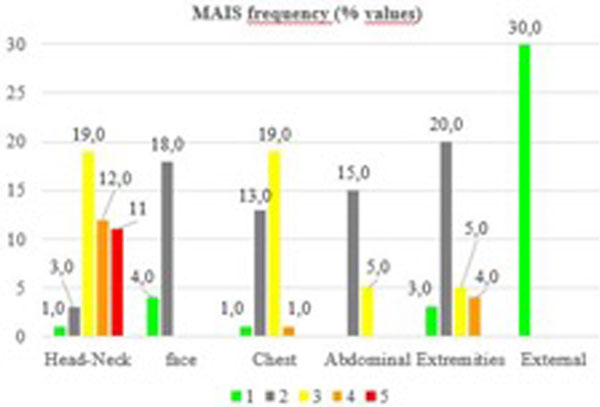
**Frequency of lesions**.

The average ISS is 21.5 (SD 10.9). Cars are the most involved in serious urban VRU crashes. Car-to-pedestrian crashes are the most frequent (50%). The impact speed is always over 40 km/hour (Table 1). The head and face are the most frequently injured part (48% of the 571 injuries collected), followed by lower extremities (15%). In terms of maximum AIS (MAIS), the head is the most severely injured region with 42% of MAIS 3+ (Figure 1).

**Table 1:** Accident dynamics: medium speed (km/hour)

Dynamic	*N *injured	Speed (SD)
Car vs. pedestrian	29	41.1 (13.9)
Motorcycle vs. pedestrian	7	40.6 (4.3)
Car vs. cyclist	10	40.7 (15.9)
Other	6	49.0 (5.4)

## Conclusion

The head is still the most frequently and severely injured region. The severity of injuries increases in the most rigid part of the car. Improving VRUs' safety devices (active and passive) to reduce impact speed and severity of the primary impact has to be the next step.
